# Bone marrow dendritic cells deficient for CD40 and IL-23p19 are tolerogenic *in vitro*

**DOI:** 10.22038/IJBMS.2020.36160.8615

**Published:** 2020-03

**Authors:** Tahereh Kalantari, Bogoljub Ciric, Eskandar Kamali-Sarvestani, Abdolmohamad Rostami

**Affiliations:** 1 Diagnostic Laboratory Sciences and Technology Research Center, School of Paramedical Sciences, Shiraz University of Medical Sciences, Shiraz, Iran; 2 Department of Laboratory Sciences, School of Paramedical Sciences, Shiraz University of Medical Sciences, Shiraz, Iran; 3 Department of Neurology, Thomas Jefferson University, Philadelphia, PA 19107, USA; 4 Immunology Department, Shiraz University of Medical Sciences, Shiraz, Iran

**Keywords:** CD40, CD40KO, IL-23, IL-23p19KO, Tolerogenic DC

## Abstract

**Objective(s):**

In addition to pro-inflammatory role, dendritic cells (DCs) can also be anti-inflammatory when they acquire tolerogenic phenotype. In this study we tested the role of CD40 and IL-23p19 in antigen presenting function of bone marrow-derived DCs (BMDCs) by comparing BMDCs derived from CD40 knockout (CD40KO-DCs) and IL-23p19 (IL-23p19KO-DCs) knockout mice with those from C57BL/6 mice (Cont-DCs). We have focused on CD40 and IL-23, as these molecules have well established pro-inflammatory roles in a number of autoimmune and inflammatory diseases.

**Materials and Methods:**

The expression of maturation markers MHC II and co-stimulatory molecules CD40, CD80, and CD86 was analyzed by flow cytometry, while the expression of CD40 and IL-23p19 was measured by RT-PCR. The capacity of BMDCs to activate CD4+ T cells was evaluated by 3H-thymidine incorporation, and the capacity of BMDCs to uptake antigen was evaluated using fluorescent OVA and flow cytometry.

**Results:**

The lack of CD40 or IL-23p19 had no effect on uptake of FITC-OVA by the DCs, confirming their immature phenotype. Moreover, CD40KO-DCs had significantly reduced capacity to stimulate proliferation of CD4+ T cells. CD4+ T cells activated by CD40KO-DCs and IL-23p19KO-DCs produced significantly less IFN-γ (*P*-value ≤0.05), while CD4+ T cells stimulated by IL-23p19KO-DCs produced less GM-CSF and more IL-10 than Cont-DCs.

**Conclusion:**

This study shows that CD40KO-DCs and IL-23p19KO-DCs have a marked tolerogenic potency *in vitro*. Future *in vivo* studies should determine if and to what extent DCs lacking CD40 or IL-23 have a potential to be useful in therapy of autoimmune inflammation.

## Introduction

Dendritic cells (DCs) have the capacity to either initiate and perpetuate immune responses as professional antigen-presenting cells (APCs), or to potently dampen immune responses if they acquire tolerogenic phenotype (1-3). The ability of DCs to activate T cell responses depends on three signals: 1. peptide-major histocompatibility complex (MHC) complexes (signal 1); 2. co-stimulatory molecules (signal 2); and 3. cytokine production (signal 3). These three signals collectively provide a complete stimuli for T cell activation and differentiation (4). However, depending on the nature of the above signals, DCs can either fail to initiate new T cell response, or they can actively suppress ongoing response through various mechanisms, such as the induction of Treg cells (4). Tolerogenic DCs express fewer co-stimulatory molecules and pro-inflammatory cytokines (5, 6), while up-regulating inhibitory cell-surface molecules and producing greater levels of anti-inflammatory cytokines such as IL-10 (6) and TGF-β (7). 

Since their discovery in 2005, Th17 cells took a central role as mediators of autoimmunity in a number of diseases, such as type 1 diabetes, rheumatoid arthritis, and experimental autoimmune encephalomyelitis (EAE), an animal model of multiple sclerosis (MS) (8-10). It has been shown that CD40, a pivotal DC costimulatory molecule, is critical to both DC maturation and Th17 cell development (11, 12). Furthermore, IL-23, a heterodimeric cytokine formed by pairing of IL-23p19 and IL-12/23p40 subunits, which is secreted by DCs, is essential to the development of pathogenic Th17 cell responses and autoimmune diseases, such as EAE and rheumatoid arthritis diseases (13, 14). Several studies have shown that suppression of CD40 or IL-23 production by DCs prevents development of Th1/Th17 responses and autoimmunity (13-17). In our previous study, we found that BMDCs with lentiviral-driven double knockdown of CD40 and IL-23p19 had tolerogenic effect due to down-regulation of CD40, lower expression of IL-6 and IL-12 and increased IL-10 production (18). In the present study, we extend these findings by testing DCs that completely lack CD40 and IL-23p19 due to genetic knockout. IL-10-treated DCs were used for comparison, as well established model for tolerogenic DCs (19). Overall, the lack of CD40 led to significant but moderate reduction in proliferation of CD4^+^ T cells, while IFN-γ production was markedly reduced. The lack of IL-23p19 led to reduction in GM-CSF production and an increase in IL-10 production. 

## Materials and Methods

***Mice***

C57BL/6, CD40 KO, and 2D2 mice were purchased from Jackson Laboratory (Bar Harbor, ME, USA). 2D2 mice are transgenic for T cell receptor (TCR) specific for myelin oligodendrocyte glycoprotein peptide (MOG_35-55_) and CD40 KO mice on C57BL/6 background were homozygous for the targeted mutation. CD40 homozygous mutant mice exhibit impaired immunoglobulin class switching and germinal center formation. IL-23p19 KO mice on C57BL/6 background were homozygous for the targeted mutation and obtained from NIH: MMRRC. All animal studies were carried out with approval of the Institutional Animal Care and Use Committee of Thomas Jefferson University.

***Generation of mouse BMDCs***

BMDCs were generated from BM progenitors by cutting both ends of the femur and tibia bones with scissors and flushing out the marrow using RPMI 1640 (20). After passing BM cell suspension through nylon mesh (100 µm), the cells were washed by centrifuging at 1500 rpm for 4 min at 4 ^°^C. The supernatant was removed and 2 ml RBC lysis buffer was added to the cell pellet. BM cells (6x10^6^) were plated and incubated at 37 ^°^C in 10 cm bacterial petri dish (low binding plates) for 3 days using complete RPMI medium supplemented with 10% FBS (CM10), L-glutamine (4 mM), penicillin (100 μg/ml), streptomycin (100 μg/ml), 50 μM 2-mercaptoethanol, and 10 ng/ml GM-CSF. Non-adherent and semi-adherent cells were collected from the petri dishes on days 3, 6, and 9 using 0.025% Trypsin-EDTA solution. BM cell cultures were pulsed for 24 hr with LPS (20 ng/ml; Sigma-Aldrich) on day 10, for the process of DC maturation. The BM cells were cultured for 2 additional days in CM10 containing 10 ng/ml GM-CSF and 30 ng/ml IL-10 to be differentiated into immature IL-10 DCs (21).

***Flow cytometric analysis***

Cultured cells were washed, suspended at 1x10^6 ^in 100 µl of PBS containing 1% FBS, 0.15% sodium azide, and 2 mM EDTA. To block Fc receptor, BMDCs were incubated with anti-CD16/32 antibody for 15 min on ice. The cells were then stained for 30 min at 4 ^°^C with conjugated rat anti-mouse CD11c, MHC class II (IA/IE), CD80 and CD86 or the appropriate isotype controls. All antibodies were purchased from BD Biosciences (San Jose, CA, USA). Stained cells were analyzed with BD fluorescence activated cell sorter (FACS) Aria (BD Biosciences) using BD FACSDiVa software (BD Biosciences).

***RNA isolation, cDNA synthesis and quantitative real time polymerase chain reaction (qRT–PCR)***

According to the manufacturer’s instructions (Qiagen, Calencia, CA, USA) total RNA was extracted from samples using the RNeasy kit and then cDNA synthesis was done (Applied Biosystems, Grand Island, NY, USA). Quantitative PCR was carried out for each sample in a final reaction mixture of 20 μl containing 10 μl TaqMan® Universal PCR Master Mix (Applied Biosystems), 9 μl cDNA and 1 μl of each primer. 6-Carboxyfluorescein (FAM)-labeled primers were used for genes IL-23p19 (IL-23a, Mm00518984_m1), CD40 (Mm00441891_m1) and 18S ribosomal rRNA endogenous control (Hs99999901_s1). The PCR was performed with the following conditions: the sample mixture was heated to 95 ^°^C and held for 10 sec as an initial denaturation step, followed by 40 cycles of 5 sec at 95 ^°^C denaturing temperature, 15 sec at 55 ^°^C annealing temperature and 1 min at 60 ^°^C extension temperature on the sequence detection system 7000 (Applied Biosystems).

***Cytokine measurement and antigen uptake assays***

Levels of interferon (IFN)-**γ**, IL-17, IL-12p70, IL-23, IL-6, IL-10, and GM-CSF in cell culture supernatants were measured by ELISA, according to the manufacturer’s instructions (R&D Systems, Minneapolis, MN, USA; and Biolegend Systems, San Diego, CA, USA). To check the antigen uptake capacity, 10×10^5 ^of DCs were incubated with ovalbumin (OVA)-FITC at a final concentration of 2 μg/ml. Control tubes were kept on ice and sample tubes were incubated at 37 ^°^C for 30 min. After washing, uptake of OVA-FITC was determined by flow cytometry.

***Statistical analysis***

Gene expression, cytokine levels and proliferation assays were analyzed by Mann–Whitney *U*-test. Differences were considered statistically significant if *P *< 0.05. All analyses were performed using spss version 17.0 or Graphpad Prism™ version 5.0 software.

## Results

***CD40KO and IL-23p19KO BMDC have similar surface phenotype as WT BMDCs***

**Figure 1:**
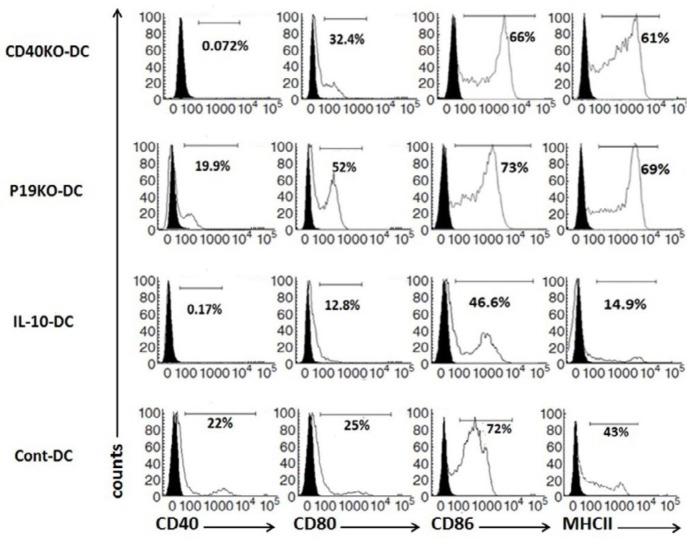
CD40KO and IL-23p19KO BMDC have similar surface phenotype as WT BMDCs. (Figure )

To characterize phenotype of BMDCs after LPS maturation, expression of MHC II, CD40, CD80 and CD86 was evaluated by flow cytometry (Figure 1). The proportion of positive cells for MHC II, CD80 and CD86 in the CD40KO and IL-23p19KO DCs was similar to Cont-DCs. IL-10-treated BMDCs had much reduced expression of CD40, CD80, and MHC II, while expression of CD86 was reduced to a lesser extent. 

***CD40KO and IL-23p19KO BMDCs have normal capacity for uptake of soluble antigen***

**Figure 2:**
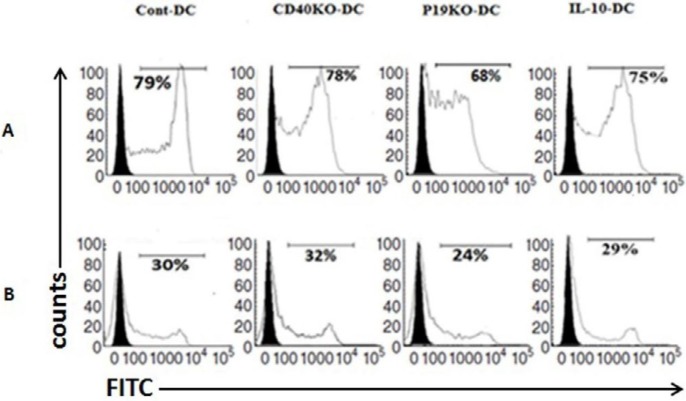
CD40KO and IL-23p19KO BMDCs have normal capacity for uptake of soluble antigen

The capacity of BMDCs to uptake soluble antigen was evaluated using FITC-OVA. As shown in Figure 2, the proportions of FITC^+^ cells among CD40KO, IL-23p19KO, IL-10-treated, and control BMDCs were similar. High endocytic capacity of CD40KO-, IL-23p19KO-, and IL-10-DCs confirmed an immature state of these DCs.

***CD40KO and IL-23p19KO BMDCs produce less pro-inflammatory cytokines***

To explore possible changes in cytokine profile of transduced BMDCs, levels of IL-23, IL-12p70 and IL-10 in culture supernatant of BMDCs were evaluated after LPS stimulation on day 10. For IL-23 measurement, supernatants were tested 6 hr after LPS stimulation, whereas IL-12p70 and IL-10 were tested 24 hr after stimulation (sec 3. A, B, C, and D). IL-10-treated DCs produced moderately lower quantities of IL-23 than Cont-DCs. CD40KO and IL-10-treated DCs produced significantly less IL-12 than Cont-DCs, while IL-23p19KO DCs also produced less IL-12 (~50%) but this reduction did not reach statistical difference. In the case of IL-6, all three DC groups (CD40KO-, IL-23p19KO-, and IL-10-

DCs) produced significantly less of this cytokine (IL-6) than Cont-DCs. IL-10-treated DCs produced significantly more IL-10 than Cont-DCs, whereas IL-23p19KO and CD40KO DCs produced similar quantities of IL-10 as Cont-DCs. 

**Figure 3:**
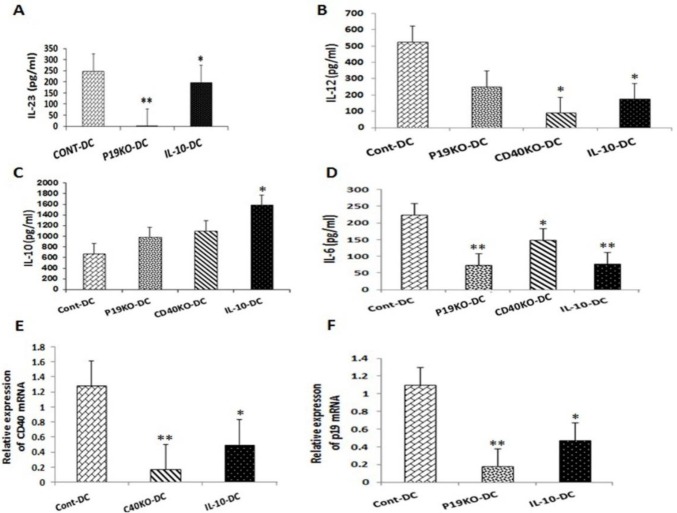
CD40KO and IL-23p19KO BMDCs produce less pro-inflammatory cytokines. Cultures of CD40KO, IL-23p19KO and IL-10-treated DCs were pulsed with LPS on day 10 of culturing and their supernatants were analyzed by ELISA for IL-23 (A), IL-12p70 (B), IL-10 (C), and IL-6 (D). Results shown are representative of two independent experiments. Levels of CD40 and IL-23p19 mRNA expression were normalized with that of 18S mRNA expression (E, F). Bars represent mean values±SEM. *P<0.05 and **P<0.01 represents statistically significant difference between test DC groups and Cont-DC group. The mean value and SEM of four independent quantitative RT-PCR experiments are shown

In addition to cytokine concentrations, mRNA levels of CD40 and IL-23p19 subunits were measured by quantitative RT-PCR in KO-derived DCs and compared to the control group. IL-10-treated DCs expressed significantly less of mRNA expression for both CD40 and IL-23p19 compared to Cont-DCs (*P*=0.025 and *P*=0.04, respectively) (Figure 3E and F)**. **

***CD40KO and IL-23p19KO BMDCs have reduced capacity to stimulate IFN-γ production by CD4*** ^+^ ***T cells***

**Figure 4.**
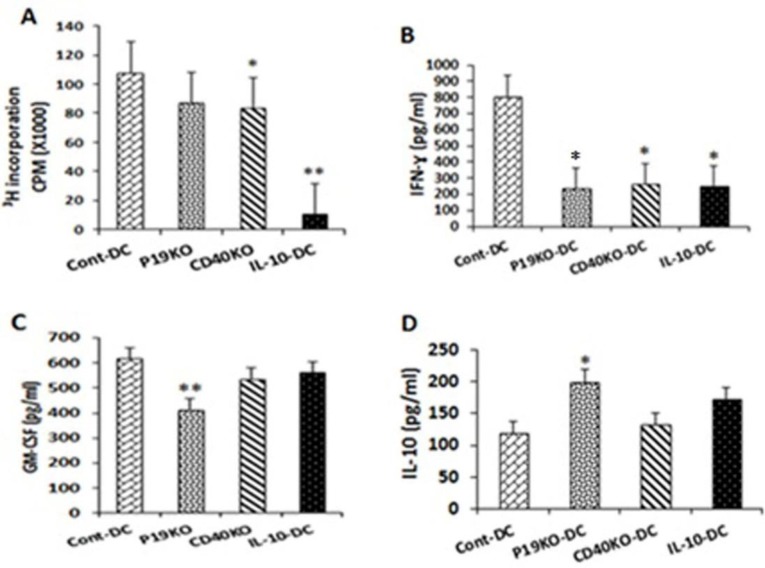
CD40KO and IL-23p19KO BMDCs have reduced capacity to stimulate IFN-γ production by CD4+ T cells. A) CD40KO, IL-23p19KO and IL-10-treated DCs were co-cultured with 2D2 CD4+ T cells at a T cell:DC ratio of 4:1 for 3 days. 3H-thymidine was added to the cultures for the last 24 h. Data are presented as the mean values of triplicate cultures±SEM. B), C), and D) IFN-γ, GM-CSF, and IL-10 were measured in the supernatants of separate CD4+ T cell-DC co-cultures by ELISA. *P<0.05 and **P<0.01 represent statistically significant difference between test co-cultures with test DCs and Cont-DCs. The results shown are representative of two independent experiments

To test the effect of CD40 and IL-23p19 KO on the capacity of DCs to mediate activation and proliferation of CD4^+^ T cells, we co-cultured CD40KO and IL-23p19KO BMDCs with CD4^+^ T cells from 2D2 mice (specific for MOG_35-55 _peptide). Even though both KO DCs exhibited modestly reduced capacity to promote T cell proliferation this reduction achieved significance only in the case of CD40KO DCs (Figure 4A). In contrast, IL-10-treated DCs failed to induce proliferation of T cells almost entirely. 

In addition to proliferation, we also tested cytokine production in DC-T cell co-cultures, as another measure of DCs’ capacity to activate T cells. In co-cultures with both types of KO and IL-10-treated DCs IFN-γ levels were sharply reduced compared with those with Cont-DCs (Figure 4B and Table 1). Furthermore, co-cultured cells produced significantly less GM-CSF (Figure 4C and Table 2) and more IL-10 (Figure 4D and Table 3) in co-culture with IL-23p19KO DCs than with the Cont-DCs.

CD40KO, IL-23P19KO, IL-10 treated-, and Cont-DCs were treated with LPS and looked over for expression of MHC II and co-stimulatory molecules. Solid histograms show background staining with isotype control monoclonal antibody, and empty histograms represent specific staining of the indicated cell-surface markers

BMDCs were incubated in medium containing 1 mg/ml of fluorescein isothiocyanate-ovalbumin (FITC-OVA) for 30 min at 37 ^°^C (A) or 4 ^°^C (B) and analyzed by flow cytometry. Solid histograms show isotype control staining, whereas empty histograms show cells staining either at 37^ °^C or 4 ^°^C

**Table 1.** Interferon (IFN)–γ production by CD4+ T cells co-cultured with KO or IL-10-treated DCs in comparison to Cont-DCs group

	**Mean ± SD** ** (pg/ml)**	** p values**
		** Cont-DCs**
** CD40KO-DCs **	262 ± 47	0.028
** P19KO-DCs**	236 ± 74	0.052
** IL-10-DCs**	252 ± 50	0.03
** Cont-DCs**	811 ± 13	1.0

KO: knockout; Cont-DCs: BMDCs derived from C57BL6 mice; Cont: control; sd: standard deviation

**Table 2.** GM-CSF production by CD4+ T cells co-cultured with KO or IL-10-treated DCs in comparison to Cont-DCs group

	**Mean ± SD** ** (pg/ml)**	** p values**
		** Cont-DCs**
** CD40KO-DCs**	537 ± 23	0.068
** P19KO-DCs**	413 ± 13	0.007
** IL-10-DCs**	562 ± 28	0.16
** Cont-DCs**	617 ± 17	1.0

KO: knockout; Cont-DCs: BMDCs derived from C57BL6 mice; Cont: control; SD: standard deviation

**Table 3:** IL-10 production by CD4+ T cells co-cultured with KO or IL-10- treated DCs in comparison to Cont-DCs

	**Mean ± SD** ** (pg/ml)**	** p values**
		** Cont-DCs**
		
** CD40KO-DCs**	132 ± 19	0.72
** P19KO-DCs**	200 ± 16	0.047
** IL-10-DCs**	173 ± 11	0.099
** Cont-DCs**	119 ± 19	1.0

KO: knockout; Cont-DCs: BMDCs derived from C57BL6 mice; Cont: control; SD: standard deviation

## Discussion

In the current study, we showed a tolerogenic effect for DCs lacking CD40 and IL-23p19. In our previous study, we showed a promising approach to make stable tolerogenic DCs by inhibiting CD40 or IL-23 gene expression in DCs with their specific shRNAs (18). In fact, to compare the effect of CD40 and IL-23p19 down-regulation by shRNA with their knockout counterparts, we used BMDCs derived from CD40KO and IL-23p19KO mice. In addition, BMDCs treated with IL-10 were used as a positive control. The next step in the study was testing tolerogenic characteristics of these BMDCs. Application of tolerogenic DC therapies for autoimmune disorders was derived from the concept that maturation conditions determine the tolerogenicity of DCs. Many different methods are available for *in vitro *generation of DCs with tolerogenic function while the most important goal in choosing the adquate method is to maintain DCs in a permanently immature state (22). Tolerogenic DCs lack second or third signals required for T cell activation. In fact, antigen presentation by tolerogenic DCs may culminate in T cell apoptosis, anergy or Treg cell differentiation (23). CD40 is one of the key co-stimulatory molecules for generating TDCs (24). CD40, a member of the tumor necrosis factor receptor (TNFR) superfamily is induced upon DC maturation (25). The original concept that immature DCs with very low levels of costimulatory molecules such as CD40 induce tolerance, whereas mature DCs with increased expression of co-stimulatory molecules induce immunity (26-29) has been revised to the new concept that tolerance is induced by immature DCs producing IL-10 (30, 31). It has been shown that exposure of *ex vivo* prepared DCs to antigens in the absence of complete maturation stimuli can prevent autoimmune diseases (9).

Our results show that after LPS activation of BMDCs, there was no significant difference in the percentage of positive cells for MHCII, CD86, and CD80 between knockout (CD40KO and IL-23p19KO) DCs.

One of the major DC functions regulated by CD40 ligation is the production of cytokines. In DCs, CD40 ligation through TRAF6 signaling leads to activation of NF-κB and production of cytokines such as IL-12p40 and IL-6 (32). It is therefore possible that suppression of CD40 expression using KO technology may prevent non-canonical NF-κB signaling and result in inhibition of IL-6 and IL-12p40 production and subsequent Th17 suppression. Consistent with this hypothesis, DCs from TRAF6 KO mice were unable to produce IL-6 or IL-12p40 in response to CD40 engagement (33). This shows a key role of NF-κB signaling in CD40 function. Based on our data from uptake assay, in the absence of LPS stimulation there was no difference between the endocytic activity of knockout DCs and Cont-DCs, as negative control, or of IL-10-DCs, as positive control. 

Our results from DCs derived from KO mice, or treated with IL-10, showed an IL-12 reduction in all DCs groups in comparison to Cont-DCs. In addition, IL-23 and IL-6 also showed significantly lower expression in all the above mentioned DC groups. Consistent with our results, several studies have demonstrated reduced production of pro-inflammatory cytokines such as IL-12 and IL-23 and increased production of anti-inflammatory cytokines such as IL-10 in tolerogenic DCs (34, 35). 

In addition, when we tested the capacity of KO-derived DC and IL-10-DC groups to stimulate MOG-specific CD4^+^ T cells, MOG-pulsed CD40KO-derived and IL-10-treated DCs failed to promote IFN-γ production by T cells. In addition**, **co-cultured cells produced a significantly lower amount of GM-CSF and a higher amount of IL-10 when co-cultured with IL-23p19KO-DCs compared to those T cells co-cultured with the Cont-DC group. Taken together, these results suggest that tolerogenic DCs can modulate antigen-specific CD4^+^ T cell responses, reducing their proliferation and polarizing their pro-inflammatory cytokine profile into an anti-inflammatory one. In our previous work, we found that DCs transduced with lentiviral vectors that contain specific shRNAs for CD40, IL-23P19, and CD40^+^IL-23P19 can be considered as TDCs, even though shRNAs for both CD40^+^IL-23P19 had the most potent TDCs (18).

## Conclusion

We found no differences in comparison with previous studies on tolerogenic DCs treated with IL-10 in expression of molecules of CD80, CD86, CD40, and MHC II which the all DCs treated with IL-10 showed lower expressions of the four surface molecules. In addition, CD40KO-DCs only showed deficient expression of CD40 with no decrease in other surface markers, while IL-23p19KO-DCs revealed no reduction in expression of CD40 surface markers compared with cont-DCs. Nevertheless, IL-23p19KO-DCs showed low secretions of pro-inflammatory cytokine IL-6, while CD40KO-DCs represented reduced levels of pro-inflammatory cytokines of IL-12 and IL-6. Although IL-10-DCs were the only DCs which produced IL-10 with a significantly increased level, CD40KO and IL-23p19KO DCs also showed higher expression of IL-10 compared to Cont-DCs. Taken together, these data show that shifting from pro-inflammatory cytokines such as IL-6, IL12, and IL-23 to anti-inflammatory cytokines like IL-10 may transform CD40KO and IL-23p19KO DCs into tolerogenic DC.

 We suggest that further* in-vivo *studies be conducted using these KO-derived DC to check their tolerogenic effect in therapy of EAE. 
